# USE OF VIDEO-BASED DATA TO INFORM PSYCHOSOCIAL INTERVENTIONS FOR PEOPLE LIVING WITH DEMENTIA

**DOI:** 10.1093/geroni/igad104.2559

**Published:** 2023-12-21

**Authors:** Gloria Hoi-Yan Wong, Jacky C P Choy

**Affiliations:** The University of Hong Kong, Hong Kong, Hong Kong; The University of Hong Kong, Hong Kong, Hong Kong

## Abstract

The pandemic has accelerated technological developments in care services. Emerging evidence suggests feasibility of delivering evidence-based psychosocial interventions such as group cognitive stimulation therapy using information and communication technology (ICT) platforms. A unique opportunity offered by ICT is the more readily available data at session level to investigate nuanced, theoretically based outcome predictors, to improve efficacy of these complex interventions. Theoretically, the cognitive and wellbeing benefits of group cognitive stimulation therapy is related to the level of person-centred engagement. As part of an ongoing randomised controlled trial involving ICT-delivered group cognitive stimulation therapy, this study examines the use of big data analytics of session behavioural data to develop predictive algorithms of cognitive and wellbeing outcomes. In 78 people living with mild to moderate dementia from 26 groups, we create automatically estimated markers of facial expression and eye movement pattern, and hand-coded markers (using time-sampling method) of engagement level and speech/verbal production information, to autobiographical information presented in the session. These markers are used to predict changes in cognitive and wellbeing outcomes. Pilot analysis using hand-coded speech sample suggested strong correlation between time-sampled engagement level with cognitive benefits, and changes in information content and fluency with cognitive benefits after the group intervention (ranging from r=0.76 to 0.97, p< 0.001). The markers we develop in this study using video-based data from ICT-delivered therapy sessions, including deep learning-based emotion recognition and eye movement analysis with hidden Markov model, allow further application in understanding the mechanisms of action in different psychosocial interventions.

